# Compositional Design of New Environmentally Friendly Geopolymer Mortar Based on Kaolin and Granite Residues

**DOI:** 10.3390/ma17225610

**Published:** 2024-11-16

**Authors:** Jeicy Ellen Alves de Brito, Alisson Mendes Rodrigues, Jucielle Veras Fernandes, Cibelle Guimarães Silva Severo, Juliana de Melo Cartaxo, Lisiane Navarro de Lima Santana, Mauro Francisco Pinheiro da Silva, Romualdo Rodrigues Menezes, Gelmires de Araújo Neves

**Affiliations:** 1 Programa de Pós-Graduação em Ciência e Engenharia de Materiais (PPG-CEMat), Universidade Federal de Campina Grande, Av. Aprígio Veloso-882, Campina Grande 58429-900, PB, Brazil; jeicyellenalves@gmail.com (J.E.A.d.B.); jucielle_fernandes@hotmail.com (J.V.F.); lisiane.navarro@ufcg.edu.br (L.N.d.L.S.); romualdo.menezes@ufcg.edu.br (R.R.M.); gelmires.araujo@professor.ufcg.edu.br (G.d.A.N.); 2 Programa de Pós-Graduação em Ciência de Materiais, Faculdade UnB Planaltina, Universidade de Brasília, Brasília 70904-910, DF, Brazil; mfps@unb.br; 3 Laboratório de Tecnologia de Materiais (LTM), Departamento de Engenharia de Materiais, Universidade Federal de Campina Grande, Av. Aprígio Veloso-882, Campina Grande 58429-900, PB, Brazil; julianamelo25@gmail.com

**Keywords:** sustainable materials, environmentally friendly materials, geopolymer mortar compositions, geopolymerization, kaolin residues, granite residues and circular economy

## Abstract

The use of industrial residues in civil construction is an exciting alternative to mitigate environmental impacts and promote the circular economy. This work developed new compositions of geopolymer mortars activated by NaOH from fine kaolin residue (RCF), coarse kaolin residue (RCG) and granite (RG). All residues were benefited and characterized by chemical analysis (X-ray fluorescence), mineralogical phases (X-ray diffraction) and granulometry (laser granulometry). Additionally, the RCF was calcined at 650 °C for 2 h (RCFC) to produce metakaolin, which is the starting point for the geopolymer reaction. A mixture of experimental designs was accomplished to evaluate the water/binder factor (W_exp_ (%)) necessary for new geopolymer mortar compositions to reach the consistency index (260 mm, ASTM C1437-15) and the effect of different curing conditions on the simple compressive strength (SCS). The geopolymeric compositions with RCFCs, pre-cured at room temperature, exhibited the highest W_exp_% values (>40%) and significant SCS, with curing conditions A and B reaching 6 MPa and 7 MPa, respectively. Such behavior can be explained by the fact that the pre-curing step at room temperature keeps the system humidity relatively high, favoring the dissolution of Si^4+^ and Al^3+^ ions and, therefore, increasing the Si/Al ratio, which positively influences the geopolymerization kinetics reaction.

## 1. Introduction

The growth of the global population and subsequent changes in human lifestyles have negatively impacted the environment, leading to the depletion of natural resources and the pollution of soil, air and water [1,2,3]. On the other hand, growing industrialization and the need for additional housing have contributed to a significant rise in cementitious material output worldwide in recent years. Also, the manufacture of clinker results in the release of a substantial quantity of carbon dioxide and other polluting compounds that contribute to the greenhouse effect, in addition to the consumption of significant amounts of natural resources (a combination of limestone, clay and other minerals) and energy [4]. In this context, geopolymeric materials made from industrial waste are promising alternatives because they not only have mechanical properties and durability comparable to or exceeding those of traditional cementitious materials, but also promote the circular economy and reduce the adverse impacts of waste disposal on social, economic and environmental dimensions [5,6,7]. In addition, geopolymers are environmentally friendly materials that reduce CO_2_ emissions by up to 80% and lower energy consumption by approximately 60% [8].

It is feasible to create a variety of materials used in civil construction from geopolymeric systems based on industrial waste, including concrete [9,10], roof tiles [11], ceramic tiles [12,13] and mortars [14]. Studies [15,16,17] on the pozzolanic activity of kaolin waste suggest that kaolin residues, in mass fractions of 5–30 wt%, can replace lime and/or Portland cement in mortar formulations. Such studies indicate that these substitutions improved the mechanical strength, durability and impermeability of mortar compositions. Granite residue can also be used in the partial replacement of cement or fine aggregate to produce gypsum mortar compositions [18]. The compressive strength, water absorption, porosity and wear resistance of such mortar formulations were assessed. Compared to normal mortars without adding residues, it was shown that adding granite residue to mortars improved their properties. The water absorption of mortars containing granite residues decreased by 20%, indicating higher durability and resistance against water penetration. In addition, when the residue was used as a sand replacement, increases in compressive strength and flexural strength were observed, reaching 2.3 MPa and 5.4 MPa, respectively. Advanced mortars incorporating multi-walled carbon nanotubes have also been studied [19,20], with the spatial structure of micropores in the cementitious composites characterized using non-destructive techniques to correlate them with experimental compression and flexural tests. In [20], the authors obtained linear responses for both compressive and flexural strengths in nano-modified prisms measuring 2 cm × 2 cm × 8 cm, with nano-additive concentrations of 0.1, 0.2, 0.3 and 0.5 wt% relative to the cement mass, respectively. As may be observed, research has already been published that has generated mortar compositions based on granite or kaolin residues. However, we are not aware of any studies that have combined these residues to create geopolymer mortar compositions activated with an alkaline NaOH solution.

With an average annual production of 7 Mt, Brazil ranks among the top five nations worldwide in granite rock production, surpassed only by countries like China, India, Turkey and Iran. Dimension stone production involves two main stages: the extraction of large granite blocks and processing (sawing and polishing). Approximately 20 to 30% of the initial block volume becomes waste during the beneficiation process [21]. Brazil also ranks among the top 10 kaolin-producing countries globally [22], with significant deposits in pegmatitic formations found in the Seridó region, located between the states of Paraíba and Rio Grande do Norte. Due to the large proportion of accessory components in primary kaolin, extraction and processing generate a tremendous quantity of waste, with 80 to 90% of the total volume of the mineral being dumped into the environment. The kaolin business produces two different forms of residues, which are collectively referred to as coarse and fine waste. A total of 70% of the residues produced by mining firms are coarse waste, which is produced when quartz is separated from kaolin ore. The beneficiation stage, which seeks to separate the fine fraction of the ore, produces a fine residue [23].

The main objective of this work is to produce geopolymer mortar compositions using residue from kaolin production and from the cutting and polishing of granite. Additionally, this study aims to investigate the percentage of water required for geopolymer mortar compositions to achieve the ideal consistency index and to evaluate the impact of various curing conditions on compressive strength through experimental designs. Ultimately, this research seeks to develop environmentally friendly products that are not only effective but also provide alternatives to reduce the improper disposal of kaolin and granite waste, as well as decrease CO_2_ emissions associated with conventional mortars.

## 2. Materials and Methods

### 2.1. Materials

The kaolin residues were donated by the Caulisa Indústria de Caulim S/A company (Juazeirinho City, Paraíba State, Brazil). Coarse kaolin residue (RCG) came from the first stage of mining processing, where the particles had an average diameter greater than 0.25 mm (D > 0.25 mm). The fine kaolin residue (RCF) was obtained from the second processing stage, called desanding, and had an average diameter greater than 0.75 mm (D > 0.75 mm). The granite residue (RG) used came from the sawing and polishing stages of the production of ornamental stones and was kindly provided by the Granfugi Mármores e Granitos company (Campina Grande City, Paraíba State, Brazil). NaOH (97%, Synth–Diadema City, São Paulo, Brazil) was used as an alkaline activator. Ammonium polyacrylate (Miracema-Noudex, Campinas, São Paulo, Brazil) were used for pH control. Table 1 shows the chemical analysis of RCF, RCG and RG.

### 2.2. Waste Beneficiation and Calcination of RCF

The RCF and RG were dried in an oven at 60 °C for 72 h, then benefited in a ball mill (TRANSMAQ, Sapucaia do Sul, Rio Grande do Sul, Brazil) (12 rpm for 40 min) and sieved (74 μm). The RCG did not need to be benefited in the mill; it was only dried in an oven and sieved (particles smaller than 1.2 mm and larger than 74 μm).

The RCG was used in the mortar as an aggregate according to the ASTM C39/C39M-21 standard [24]. RCF was used with the aim of producing metakaolin through calcination and being the precursor in the activation reaction. Therefore, after the beneficiation step, the RCF was calcined at 650 °C for 2 h, with a heating rate of 5 °C/min [15,16]. The fine calcined kaolin residue was called RCFC.

### 2.3. Characterizations of Raw Materials

Chemical analysis was performed using X-ray fluorescence spectrometry (XRF) with an EDS detector (Shimadzu, EDX 720 model, Kyoto, Japan). The granulometric analysis was performed via laser granulometry (Cilas, model 1064LD, Orléans, France). The X-ray diffraction experiments were carried out in a Shimadzu Diffractometer (model XRD-6000, Kyoto, Japan) equipped with CuKα radiation. Simple compressive strength (SCS) experiments were performed in a universal testing machine (Shimadzu, model AG-15 150 KN, Kyoto, Japan) at a rate of 5 N/s. The consistency index was measured according to ASTM C1437 [25].

### 2.4. Experimental Design

Preliminarily, the residues’ proportion in the mortar compositions used in the consistency index experiments and to evaluate the effect of curing conditions on mechanical strength was determined with the aid of the statistical design of mixture experiments, a centroid simplex lattice design augmented with a central point [26]; see Table 2. To determine the water/binder factor (W_exp_ (%)) in the mortar compositions proposed, the consistency index was measured in agreement with ASTM C1437-20 [25], where the RCFC, RCG and RG amounts were the independent variables. The experiments were performed in triplicate. Mathematical models (linear, quadratic, cubic and special cubic) were adjusted to experimental data to verify which one was more representative and predictive. To evaluate the effect of different curing conditions on SCS, the RCFC, RCG and RG were also the independent variables, but the SCS values were response variables. The experimental design and the statistical analysis of the data were carried out using the Statistica 14.0 software [27].

### 2.5. Cure Conditions

Cylindrical samples with a diameter of 2.5 cm and a height of 5.0 cm were used to evaluate the effects of curing conditions on mortar compositions. These samples were also used in SCS experiments. To obtain such samples, the RCFC, RCG and RG were dried and weighed according to the proportions defined by the experimental design. Concomitantly, a NaOH solution (40 g NaOH for 900 mL of H_2_O), according to ASTM C1260-21 [28], was prepared with 80% of the water determined by the experimental design. This solution was allowed to stand for 4 h. The other 20% of water determined by the experimental design was used to obtain an NH_4_OH solution (40 g NaOH for 900 mL of H_2_O) according to ASTM C1260-21 [28]. After that, the NaOH and NH_4_OH solutions were added to the previously weighed residues, and the system was homogenized in a mechanical mixer. The homogeneous mixture was poured into a mold (diameter of 2.5 cm and height of 5.0 cm) to obtain cylindrical samples. Then, such samples were kept under different curing conditions (see Table 3) with subsequent measurements of the SCS.

## 3. Results and Discussion

### 3.1. Chemical Analysis, Mineralogical Phases and Particle Size Distribution

Each residue used in this study was characterized to confirm the function it can perform in the compositions of the newly designed geopolymer mortars. Table 1 shows that the SiO_2_ and Al_2_O_3_ contents detected in the RCF and RCG were 52.5 wt% and 53.5 wt%, respectively. For geopolymeric systems to function, SiO_2_ and Al_2_O_3_ must be present as they tend to form aluminosilicates, which significantly impact the mechanical and microstructural properties [29,30]. As expected, SiO_2_, Al_2_O_3_, Fe_2_O_3_ and CaO contents were detected in the RG. The presence of Fe_2_O_3_ and CaO contents is related to the chemical composition of the granite, as well as the addition of grit and calcium oxide, which are often used as a lubricant and abrasive in the industrial process of cutting/sawing this material [31]. SiO_2_ and Al_2_O_3_ values in the RG were 49.4 wt% and 18.5 wt%, respectively.

**Figure 1:**
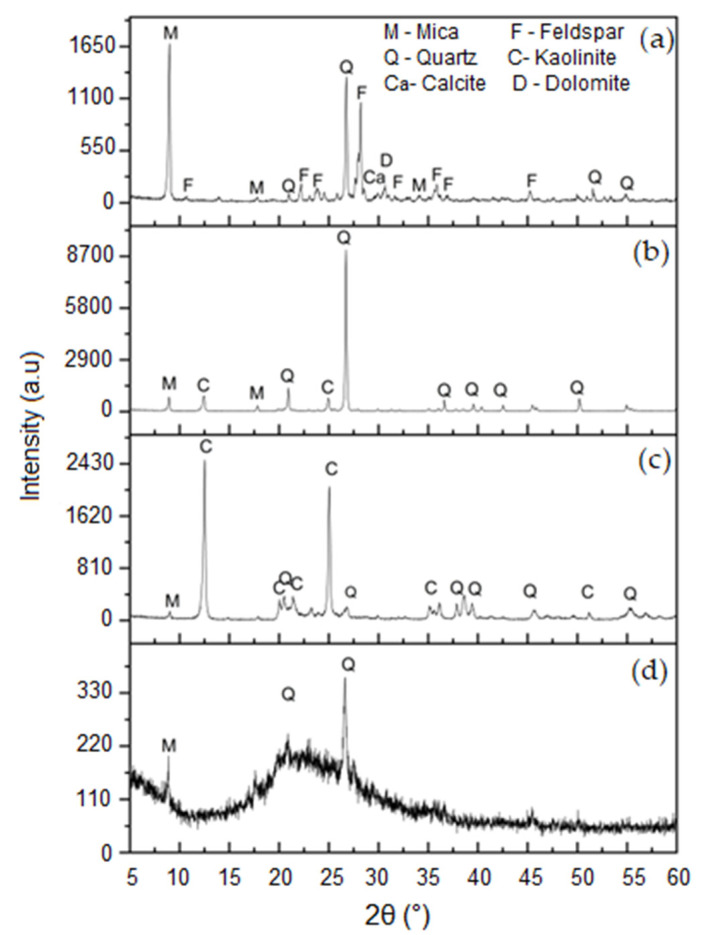
Diffractograms of RG (**a**), RCG (**b**), RCF (**c**) and RCFC (**d**).

The mineralogical analysis of the RCF, RCG and RG is shown in Figure 1a–c. The mica (JCPDS 83-1808), feldspar (JCPDS 83-1808) and quartz (JCPDS 46-1045) phases were detected in the RG (Figure 1a), indicating that they come from granitic rocks [32]. The calcite (JCPDS 47-1743) and dolomite (JCPDS: 89-5862) phases were also detected in the RG and are related to the limestone used as a lubricant and alkalinizer of the environment during the sawing process. Mica, kaolinite (JCPDS 78-2110) and quartz were the mineralogical phases identified in RCF and RCGs; see Figure 1b,c. The presence of the kaolinite phase (Si_2_O_5_Al_2_(OH)_4_) is essential for the geopolymerization reaction since it can be a source of metakaolin (Si_2_O_5_Al_2_O_2_) through a dehydroxylation reaction (Si_2_O_5_Al_2_(OH)_4_ → Si_2_O_5_Al_2_O_2_ + 2H_2_O) [33]. Therefore, to form metakaolin, the RCF was calcined at 650 °C for 2 h. This new calcined material was called fine calcined kaolin waste (RCFC). As expected, the diffractogram of the RCFC (Figure 1d) is characteristic of an amorphous material and kaolinite peaks were not detected, indicating the efficiency of calcination. The presence of amorphous metakaolin favors pozzolanic activity in mortars with the potential to influence the decrease in porosity and increase mechanical strength and durability [34,35,36].

**Figure 2:**
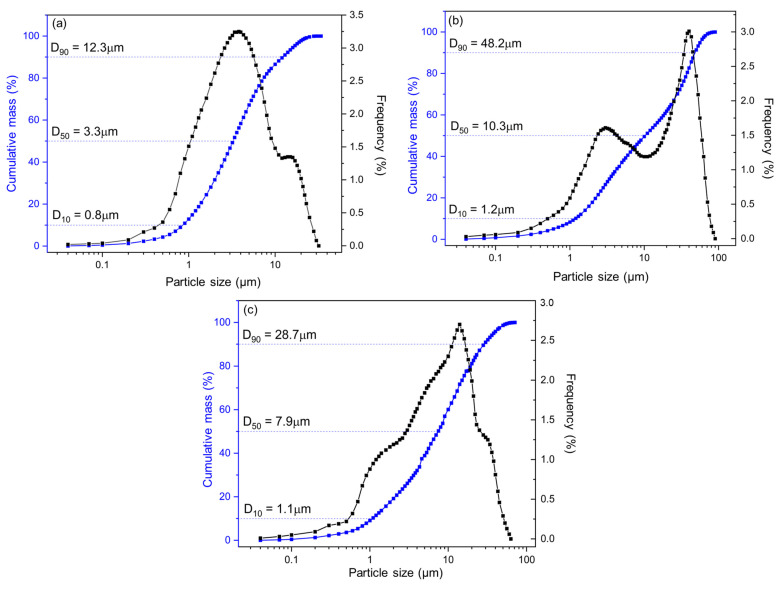
Granulometric analysis of (**a**) RCFC, (**b**) RCG and (**c**) RG.

The following experimental results were obtained considering RCFC since this would be the source of metakaolin for the mortar compositions investigated in this work. Particle size distribution curves (cumulative and frequency) of RCFC, RCG and RG are shown in Figure 2a, Figure 2b and Figure 2c, respectively. The RCFC presented a bimodal frequency curve with all particles with sizes smaller than 12.3 µm, with D_90_ = 12.3 µm, D_50_ = 3.3 µm and D_10_ = 0.8 µm. The RCG also showed a bimodal frequency curve with particles smaller than 48.2 µm, with D_90_ = 48.2 µm, D_50_ = 10.3 µm and D_10_ = 1.2 µm. The RG showed particle sizes smaller than 28.7 µm, with D_90_, D_50_ and D_10_ equal to 28.7 µm, 7.9 µm and 1.1 µm, respectively. Comparatively, RCG and RG showed a wider curve profile, with more considerable variation in particle size than RCFCs. Table 4 shows the percentage of accumulated mass for different particle size ranges and their respective mean diameters for RCFC, RCG and RG. Approximately 32% of the RCFC particles were smaller than 2 μm, while the RCG and RG were only 17.8% and 19.1%, respectively. The fraction of particles of the RCFC comprising sizes between 2 µm and 20 µm was 66.2%, while for the RCG and RG, they were 44.0% and 62.0%, respectively. The RG had the highest fraction of particles with sizes above 20 µm, followed by the RG (18.9%) and RCFC (2.2%). The identification of the metakaolin in the RCFC (see Figure 1d), plus the relatively fine granulometry, justifies the use of this residue as a precursor of the geopolymer reaction. On the other hand, the use of RCG and RG as an aggregate is primarily justified by their relatively larger granulometry.

### 3.2. Design of Mortar Compositions Containing RCFC, RCG and RG

The water/binder factor (W_exp_ (%)) necessary for each mortar composition suggested by the experimental design to reach the consistency index (260 mm [37]) is listed in Table 5. Mortar composition 1 required more water to achieve the desired consistency index (W_exp_ = 52.0%). Still, as it only contains RCFC, it is uninteresting in terms of the suggested application of RCFC, RG and RCG. For mortar composition 10, less water was needed to achieve the consistency index. However, the absence of the RCFC in this mixture might have an adverse effect on its mechanical characteristics. In actuality, the function of RCFC is to serve as a source of metakaolin, a substance that aids in the geopolymerization reaction (see Figure 1d) [34,36,38]. Since all the residues examined in this paper contribute to the mortar composition numbers 4, 5, 6 and 7, they are intriguing from a sustainable perspective. For the consistency index, the W_exp_ (%) values for mortar compositions 4, 5, 6 and 7 were 35.5%, 32.5%, 35.0%, and 43.0%, respectively. It is feasible to deduce from an investigation of the compositions of mortars 4, 5, 6 and 7 that the values of W_exp_% rise with the amount of RCFC residue.

The linear, quadratic, cubic and special cubic mathematical models were adjusted to the experimental data shown in Table 5. The most representative and predictive mathematical adjustments were chosen based on three criteria: (I) presenting *p*-values lower than 0.05, (II) presenting an F_test_ greater than 5 (F_test_ > 5) and (III) higher R^2^ values. Table 6 summarizes the results of the mathematical adjustments performed. From the viewpoint of criterion (I), no mathematical adjustment showed *p*-values above 0.05. The cubic model was disregarded because it presented an F_test_ value below 5. Both adjustments performed with the linear, quadratic and special cubic models presented F_test_ values > 5; however, the special cubic model was considered more representative and predictive because it had a higher R^2^ value (0.93). In this sense, Equation (1) represents the dependence on the amount of water necessary for the mortar compositions to reach the consistency index. In this equation, the coefficients a, b and c correspond to the percentages of the RCFC, RG and RCG, respectively. All coefficients were statistically significant at the 95% confidence level.

W_cal_ (%) = 52.75a + 32.60b + 35.30c − 12.14ab − 8.14ac − 4.80bc 59.82abc + 14.68ab(a − b) − 6.33ac(a − c) (1)

**Figure 3:**
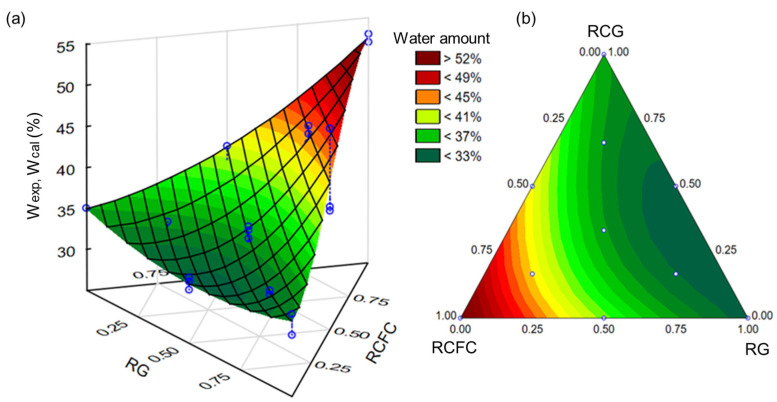
(**a**,**b**) 3D response surface plots and their respective projections onto the composition triangle were calculated for mortar compositions containing RCFC, RG and RCG.

The 3D response surface plot and its corresponding projection, derived from the mathematical fit using the special cubic model (see Equation (1)), are presented in Figure 3a,b. The trend observed in Table 5 was confirmed; mortar compositions with higher proportions of RCFCs required more water to achieve a consistency index of 260 mm. This behavior is attributed to the smaller average particle diameter of RCFC (5.0 µm, as shown in Table 4) compared to the other residues, resulting in a larger surface area. Generally, mortar compositions containing at least 60% RCFC required over 40% water by volume to reach the desired consistency. Conversely, the lowest water volumes (>35%) were necessary for mortar compositions containing over 65% RG. It is known that higher water content tends to reduce mechanical strength. This introduces an interesting element to the study, as the mortar compositions requiring the highest water content to reach the target consistency are those with a greater RCFC proportion, which is critical for the geopolymerization reaction.

### 3.3. Influence of Different Curing Conditions on SCS

The influence of curing conditions (A, B, C, D and E; see Table 3) and alkaline activation (NaOH) on SCS values was evaluated for mortar compositions suggested by the experimental design, see Table 7.

The linear, quadratic, cubic and special cubic mathematical models were also fitted to the data shown in Table 8, and the most representative and predictive fits were also chosen according to *p*-values below 0.05, F_test_ values above 5 and higher R^2^ values. For some cure conditions (C, D and E), it was not possible to adjust all mathematical models. For cure condition A, the special cubic model was not considered predictive since the *p*-value was above 0.05. Considering an F_test_ > 5 and higher R^2^ values, we conclude that the cubic model is more predictive for mortar compositions cured under conditions A and D while the special cubic model is more predictive for mortar compositions cured under conditions B, C and E. The equations describing the most predictive fit for cures A, B, C, D, and E are listed in Table 9.

**Figure 4:**
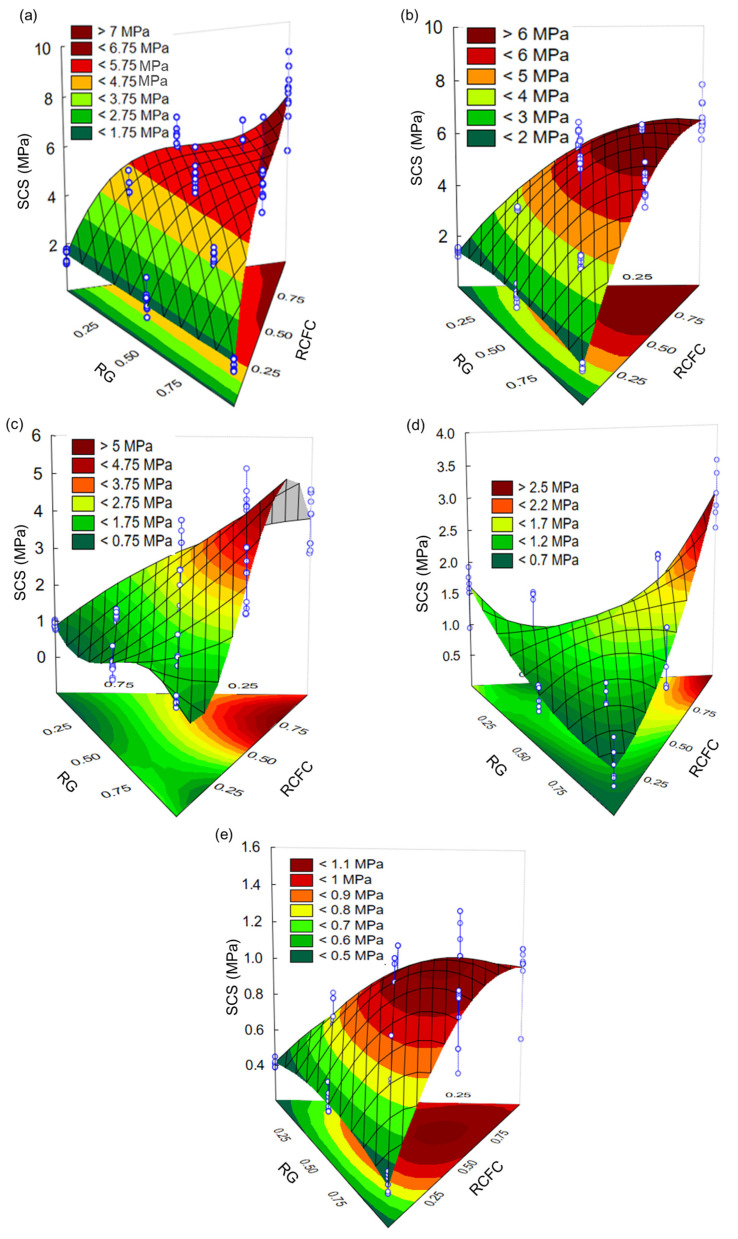
Effect of curing conditions on the SCS values of NaOH-activated mortar compositions containing RCFC, RCG and RG, (**a**) cure A; (**b**) cure B; (**c**) cure C; (**d**) cure D and (**e**) cure E.

The effect of curing conditions on SCS values in mortar compositions containing RCFC, RCG and RG is shown in the 3D response surface plots; see Figure 4a–e. Such graphs were constructed from the equations shown in Table 9. From the viewpoint of composition, regardless of the curing conditions used, the highest SCS values were observed for mortars containing higher levels of RCFC. As shown in Figure 1d, the RCFC is a source of metakaolin, which, due to factors such as a high specific surface, a high chemical reactivity and being a source of aluminosilicate, plays a key role in increasing the geopolymerization reaction kinetics [29,30,39]. From the perspective of curing conditions, the highest SCS values were observed for mortar compositions kept under curing conditions A and B (>7 MPa and >6 MPa, respectively). These curing conditions have, typically, a pre-curing stage of 72 h at room temperature, and only then are they cured for 72 h at different temperatures, that is, 60 °C and 100 °C for curing conditions A and B, respectively. The pre-curing step at room temperature, due to the availability of water, allowed for a more pronounced dissolution of Si^4+^ and Al^3+^ ions, increasing the Si/Al ratio. The Si/Al ratio is one of the factors that affect the SCS of geopolymers [40,41,42]. Furthermore, water molecules and available OH^-^ ions are also consumed in the first step of geopolymerization [43,44].

Comparatively, mortar compositions cured under conditions C and D (Figure 4c,d) showed lower SCS values than those maintained under curing conditions A and B (Figure 4a,b). As commented on before, this occurred due to the absence of a pre-cure step at room temperature, which decreased the concentration of Si^4+^ and Al^3+^ ions available for the polymerization reaction. Besides that, the fact that the samples were taken directly to the oven at a temperature above room temperature (60 °C and 100 °C for curing conditions C and D, respectively) contributed significantly to the decrease in humidity and, therefore, in the water amount and, thus, the OH^-^ ions available for the geopolymerization reaction. The lowest SCS values (<1.1 MPa) were measured from mortar compositions cured for 240 h at room temperature (cure E). Here, again, the absence of a curing step above room temperature appears to have significantly influenced the kinetics of the geopolymerization reaction. In fact, in studies [5,6,7,14,45,46], regarding the influence of temperature and curing time on the strength of geopolymers, it was found that curing at room temperature is not feasible due to the long time required for the beginning of the geopolymerization reaction to occur.

### 3.4. Recommendations for Future Studies

For future studies, it would be valuable to further explore a few points, including the use of alternative alkaline activators, such as potassium hydroxide (KOH) and sodium silicate (Na_2_SiO_3_); investigating the aging behavior and durability of geopolymer products; examining the effect of water curing on geopolymer performance; and assessing the impact of RCG calcination on the properties of the resulting geopolymer products.

## 4. Conclusions

New geopolymer mortar compositions were developed using residue from the kaolin processing industry and granite sawdust. According to XRD analysis, metakaolin was successfully produced through the calcination of RCF, yielding RCFC, which served as a precursor in the geopolymer reaction. Geopolymer mortar compositions with higher concentrations of RCFC required a greater water percentage to achieve the desired consistency, likely due to RCFC’s larger surface area from its smaller average particle diameter (5.0 µm). Regarding the effect of curing conditions on SCS, the inclusion of a pre-curing step at room temperature was essential for achieving higher SCS values (6 MPa and 7 MPa for curing conditions A and B, respectively). This improvement is likely due to moisture facilitating the dissolution of Si^4+^ and Al^3+^ ions, which in turn accelerated the geopolymerization reaction. Overall, this study enabled the development of sustainable and environmentally friendly mortar formulations, offering an alternative to conventional materials. Additionally, this approach supports the efficient reuse of industrial residues, contributing to a reduction in the environmental impact.

## Figures and Tables

**Table 1:** Chemical composition of RCF, RCG and RG.

Components	RCF (wt%)	RCG (wt%)	RG (wt%)
SiO_2_	52.5	53.5	49.4
Al_2_O_3_	45.0	43.4	18.5
K_2_O	0.8	1.4	3.4
MgO	1.1	1.0	4.1
Fe_2_O_3_	0.4	0.5	12.5
SO_3_	--	--	0.4
CaO	--	--	6.3
Na_2_O	--	--	2.6
TiO_2_	--	0.1	2
P_2_O_5_	--	--	0.6
Others	0.2	0.1%	0.2
Si/Al	1.05	0.899	2.32

**Table 2:** Mortar compositions containing RCFC, RCG and RG were suggested by the experimental design of centroid simplex.

Mortar Compositions	Residue Proportions (%)		
	RCFC	RCG	RG
1	100	0	0
2	0	100	0
3	0	0	100
4	33	33	33
5	16.67	16.67	66.66
6	16.67	66.66	16.67
7	66.66	16.67	16.67
8	50	50	0
9	50	0	50
10	0	50	50

**Table 3:** Curing conditions under which mortar compositions containing RCFC, RCG, and RG were maintained.

	Cure Conditions
A	72 h at room temperature and 72 h at 60 °C
B	72 h at room temperature and 72 h at 100 °C
C	72 h at 60 °C
D	72 h at 100 °C
E	240 h at room temperature

**Table 4:** The mean diameter and granulometric particle size distribution of RCFC, RCG and RG.

Residues	Average Diameter (µm)	(x < 2 µm)	(2 µm < x < 20 µm)	(x > 20 µm)
RCFC	5.0	31.6	66.2	2.2
RCG	11.8	17.8	44.0	38.2
RG	18.8	19.1	62.0	18.9

**Table 5:** W_exp_ (%) values measured for the mortar compositions containing the RCFC, RCG and RG, as suggested by the experimental design.

Mortar Compositions	Residues			W_exp_ (%)
	RCFC	RCG	RG	
1	100	0	0	52.0
2	0	100	0	35.0
3	0	0	100	32.5
4	33	33	33	35.5
5	16.67	16.67	66.66	35.0
6	16.67	66.66	16.67	32.5
7	66.66	16.67	16.67	43.0
8	50	50	0	41.0
9	50	0	50	39.5
10	0	50	50	32.5

**Table 6:** F_test_, R^2^ and *p*-values obtained from the mathematical adjustment of the linear, quadratic, cubic and special cubic models to the experimental data in Table 5.

Mathematical Models	F_test_	R^2^	*p*-Values
Linear	74.9	0.83	0.000
Quadratic	6.1	0.89	0.003
Cubic	0.95	0.91	0.400
Special Cubic	5.9	0.93	0.022

**Table 7:** SCS values were measured from the NaOH-activated mortar compositions maintained under curing conditions A, B, C, D and E.

Mortar Compositions	Residues			NaOH (Alkaline Activator)				
	RCFC	RCG	RG	Cure A	Cure B	Cure C	Cure D	Cure E
1	100	0	0	7.1	5.7	3.97	3.45	0.57
2	0	100	0	1.77	1.57	0.77	1.66	0.45
3	0	0	100	1.93	1.55	1.8	1.19	0.46
4	33	33	33	6.97	6.19	1.86	1.27	0.88
5	16.67	16.67	66.67	3.98	2.97	2.64	1.01	0.65
6	16.67	66.67	16.67	3.15	3.15	1.63	1.79	0.74
7	66.67	16.67	16.67	4.43	5.79	4.6	2.3	1.21
8	50	50	0	5.47	4.7	2.45	0.67	0.59
9	50	0	50	5.48	5.07	3.77	1.19	0.69
10	0	50	50	2.11	1.81	1.25	0.96	0.63

**Table 8:** Shows the F_test_, R^2^ and *p* values obtained from the adjustment of the linear, quadratic, cubic and special cubic models to the data in Table 7.

Cure	Mathematical Model	Test F	R^2^	*p*-Value
A	Linear	135	73	0.000
	Quadratic	23	84	0.000
	Cubic	13	87	0.000
	Special Cubic	4	84	0.052
B	Linear	144.7	75	0.000
	Quadratic	74	90	0.000
	Cubic	8	92	0.005
	Special Cubic	20	92	0.000
C	Linear	26	46	0.000
	Quadratic	13.4	67	0.000
	Special Cubic	28	80	0.000
D	Linear	133	73	0.000
	Quadratic	9	77	0.000
	Cubic	24	85	0.000
E	Linear	50	49	0.000
	Quadratic	33	74	0.000
	Special Cubic	13.3	80	0.000

**Table 9:** Models and their respective equations show better dependence between the compressive strength, curing condition and alkaline activator (NaOH) for mortar compositions containing RCFC, RCG and RG.

Cure	Model	Regression Equation	
A	Cubic	SCS (MPa) = 7.05x + 1.55y + 1.76z + 4.69xy + 6.44xz + 18.71xyz − 10.84xy(x − y) − 8.12xz(x − z)	(2)
B	Special cubic	SCS (MPa) = 6.45x + 1.38y + 1.40z + 6.27xy + 7.26xz + 1.71yz + 20.20xyz	(3)
C	Special cubic	SCS (MPa) = 2.99x + 1.63y +0.70z − 6.14xy − 2.31xz − 1.36yz + 23.46xyz+	(4)
D	Cubic	SCS (MPa) = 3.85x + 0.857y + 1.89z + 2.42xy + 2.12xz + 16.68xz(x − z) − 4.69yz(y − z)	(5)
E	Special cubic	SCS (MPa) = 0.97x + 0.42y + 0.419z + 0.80xy + 1.12x z+ 0.374yz + 5.16xyz	(6)

## Data Availability

The original contributions presented in the study are included in the article, further inquiries can be directed to the corresponding author.
